# Prevention of hepatic encephalopathy by administration of rifaximin and lactulose in patients with liver cirrhosis undergoing placement of a transjugular intrahepatic portosystemic shunt (TIPS): a multicentre randomised, double blind, placebo controlled trial (PEARL trial)

**DOI:** 10.1136/bmjgast-2020-000531

**Published:** 2020-12-28

**Authors:** K de Wit, J J Schaapman, F Nevens, J Verbeek, S Coenen, F J C Cuperus, M Kramer, E T T L Tjwa, N Mostafavi, M G W Dijkgraaf, O M van Delden, U H W Beuers, M J Coenraad, R B Takkenberg

**Affiliations:** 1 Gastroenterology and Hepatology, Amsterdam UMC, University of Amsterdam, Amsterdam Gastroenterology Endocrinology Metabolism, Amsterdam, The Netherlands; 2 Gastroenterology and Hepatology, Leiden University Medical Center, Leiden, The Netherlands; 3 Gastroenterology and Hepatology, University Hospitals KU Leuven, Leuven, Belgium; 4 Gastroenterology and Hepatology, Erasmus University Medical Center Rotterdam, Rotterdam, The Netherlands; 5 Gastroenterology and Hepatology, University Medical Center Groningen, Groningen, The Netherlands; 6 Division of Gastroenterology and Hepatology, Department of Internal Medicine, Maastricht University Medical Center, Maastricht, The Netherlands; 7 Gastroenterology and Hepatology, Radboud University Medical Center, Nijmegen, The Netherlands; 8 Biostatistics Unit, Department of Gastroenterology and Hepatology, Amsterdam UMC, University of Amsterdam, Amsterdam Gastroenterology Endocrinology Metabolism, Amsterdam, The Netherlands; 9 Epidemiology and Data Science, Amsterdam UMC, University of Amsterdam, Amsterdam, The Netherlands; 10 Interventional Radiology, Amsterdam UMC, University of Amsterdam, Amsterdam, The Netherlands

**Keywords:** hepatic encephalopathy, liver cirrhosis, portal hypertension

## Abstract

**Introduction:**

Cirrhotic patients with portal hypertension can suffer from variceal bleeding or refractory ascites and can benefit from a transjugular intrahepatic portosystemic shunt (TIPS). Post-TIPS hepatic encephalopathy (HE) is a common (20%–54%) and often severe complication. A prophylactic strategy is lacking.

**Methods and analysis:**

The Prevention of hepatic Encephalopathy by Administration of Rifaximin and Lactulose in patients with liver cirrhosis undergoing placement of a TIPS (PEARL) trial, is a multicentre randomised, double blind, placebo controlled trial. Patients undergoing covered TIPS placement are prescribed either rifaximin 550 mg two times per day and lactulose 25 mL two times per day (starting dose) or placebo 550 mg two times per day and lactulose 25 mL two times per day from 72 hours before and until 3 months after TIPS placement. Primary endpoint is the development of overt HE (OHE) within 3 months (according to West Haven criteria). Secondary endpoints include 90-day mortality; development of a second episode of OHE; time to development of episode(s) of OHE; development of minimal HE; molecular changes in peripheral and portal blood samples; quality of life and cost-effectiveness. The total sample size is 238 patients and recruitment period is 3 years in six hospitals in the Netherlands and one in Belgium.

**Ethics and dissemination:**

This study protocol was approved in the Netherlands by the Medical Research Ethics Committee of the Academic Medical Centre, Amsterdam (2018-332), in Belgium by the Ethics Committee Research UZ/KU Leuven (S62577) and competent authorities. This study will be conducted in accordance with Good Clinical Practice guidelines and the principles of the Declaration of Helsinki. Study results will be submitted for publication in a peer-reviewed journal.

**Trial registration numbers:**

ClinicalTrials.gov (NCT04073290) and EudraCT database (2018-004323-37).

## Introduction

### Portal hypertension and TIPS

Liver cirrhosis is the most common cause of portal hypertension (PH). Complications of PH include (refractory) ascites and oesophageal or gastric variceal bleedings. These complications are severe and result in hospital admissions and decreased survival. Moreover, cirrhotic patients undergo physical deterioration due to undernutrition and muscle wasting and are physically frail.^1^

A transjugular intrahepatic portosystemic shunt (TIPS) reduces PH, by decompression of the portal system through an artificial shunt from a intrahepatic portal vein to a hepatic vein. TIPS can be considered as therapy for refractory ascites and variceal bleeding. TIPS treatment for refractory ascites has a response rate, defined as improvement of ascites, of up to 85%.^2 3^ A meta-analysis showed improved survival of cirrhotic patients receiving a (uncovered) TIPS, with a decreased risk of refractory ascites and hepatorenal syndrome.^4^ However, these patients have an increased risk of hepatic encephalopathy (HE). Improvement of survival was confirmed in a recent randomised controlled trial (RCT): 1-year transplant-free survival was significantly higher in patients who received TIPS (93%) compared with patients who were treated with large volume paracentesis (LVP) and albumin infusion (52%) and suggested that TIPS should be the first line of treatment of refractory ascites.^5^

Another consequence of PH is the development of gastric or oesophageal varices. One-third of the patients who are diagnosed with liver cirrhosis and are compensated have oesophageal varices, and in patients presenting with ascites, this is the case in up to 60%.^6^ Seventy per cent of all upper gastrointestinal bleeding episodes in patients with cirrhosis are caused by variceal bleeding.^6^ In patients with multiple bleeds, high risk of treatment failure (recurrence of variceal bleed), or increased mortality risk (Child Pugh C (<11 points) liver cirrhosis), a TIPS is a highly effective intervention (94%) to control bleeding.^7^ Hence, TIPS is a very effective intervention to treat complications of PH.^2 3 7^ However, also these patients are at risk for post-TIPS HE.^8–10^

### Hepatic encephalopathy

HE is a common complication in patients with liver cirrhosis and is associated with profound loss of quality of life. HE is a brain dysfunction caused by liver insufficiency and/or portosystemic shunting; it manifests as a wide spectrum of neurological or psychiatric abnormalities ranging from subclinical alterations to coma.^11^ HE is classified using the West-Haven criteria: minimal HE (MHE), covert HE (grade I) or overt HE (OHE, grades II–IV).^12^ MHE is subtle cognitive impairment that is difficult to detect clinically and only by psychometric testing. The psychometric hepatic encephalopathy score (PHES) and simplified one min animal naming test (S-ANT1) are two validated instruments to measure MHE.^13 14^ PHES exists of a line tracing test, digit symbol test, serial dotting test and a number connection test.^13^ S-ANT1 is a 1 min test in which patients name as many animals as possible. The outcome of this test was validated to score MHE.^14^ OHE presents with a varying severity and symptoms as disorientation, motor dysfunctions, abnormalities in behaviour, intellectual functions and consciousness. Thirty to forty-five per cent of patients with liver cirrhosis develop OHE during the course of their disease.^15^ HE leads to a prolonged duration of hospital admissions, an increased number of primary care contacts, an impaired quality of life and increased mortality.^11 16–19^

### Treatment and prevention of OHE

The initial treatment of OHE is non-absorbable disaccharides like lactulose.^11^ The dosing of lactulose should be initiated with 25 mL of lactulose every 12 hours and the dose should be titrated to achieve two soft or loose bowel movements per day.^11^ The working mechanism of lactulose is not fully understood, but it is assumed that the prebiotic effects and acidifying nature of lactulose have an additional benefit beyond the laxative effect.^20 21^

There is currently no treatment registered for primary prevention of HE. Secondary prevention of HE can be achieved by combination therapy of lactulose with rifaximin, a poorly absorbed antibiotic. In the Netherlands, rifaximin has been approved since 2016 and will be reimbursed only to prevent a third (or following) episode of OHE.^22^

### Post-TIPS HE

HE is a common and often severe complication after TIPS placement. Incidence of new onset OHE or worsening of MHE after TIPS is approximately 20%–54%.^7–10^ Risk factors for post-TIPS HE are mainly described for uncovered stents in retrospective studies. Extensive prospective evidence is lacking and little evidence is available for covered stents. Prior HE episodes, increased CP score and increased age are possible risk factors, but there is no consensus about the exact limits.^23^ In patients with variceal bleeding, a TIPS diameter of 8 mm can decrease the incidence of post-TIPS HE compared with 10 mm.^24^

The International Club of Ascites published their sixth position paper on PH in 2015.^25^ Both this group of international experts in PH as well as the European Association for the Study of the Liver (EASL)/American Association for the Study of Liver Diseases (AASLD) practice guidelines have prioritised studies on treatment and prevention of (post-TIPS) HE on the research agenda because of the lack of strong evidence regarding primary prevention.^11 25^ Besides a missing strategy to prevent HE after TIPS placement, it is currently not possible to predict whether patients will suffer from post-TIPS HE. Moreover, possible biomarkers currently are lacking.

### Prevention of post-TIPS HE

An RCT, Riggio *et al*, showed that rifaximin (1200 mg/day) alone or lactitol (60 mL/day) alone did not prevent HE after TIPS placement.^26^ It is unclear whether polytetrafluoroethylene covered stents or bare metal stents were used. Although not fully published yet, a more recent RCT, Bureau 2019, does show a positive effect of rifaximin on post-TIPS HE. In this trial, rifaximin 1200 mg/day was started 15 days before TIPS placement until 6 months post-TIPS.

The combination of rifaximin and lactulose has never been tested to prevent post-TIPS HE. This might prove to be a more effective strategy, as it has been shown to be effective in the prevention of recurrent HE and is current standard of care.^27^ In this particular RCT—both in patients with and without TIPS—a reduction of 58% in episodes of HE was realised, and a 50% reduction in the risk of hospitalisation was achieved.^27^ Two other double-blind RCTs confirmed that combination of lactulose and rifaximin decreased the risk of HE.^28 29^ Apart from inhibition of intestinal bacterial RNA synthesis, effects of rifaximin on intestinal cells can be observed within 24 hours. Intestinal barrier function is improved, and there is an upregulation of enzymes involved in detoxification.^30 31^ Based on the effectiveness of lactulose and rifaximin, we hypothesise that the combination of lactulose and rifaximin will reduce the number of patients with post-TIPS HE.

## Objectives

### Primary objective

To assess the incidence of post-TIPS OHE within the first 3 months after prophylactic administration of lactulose and rifaximin versus lactulose and placebo in patients who undergo covered TIPS placement.

### Secondary objectives

To assess:

1. Ninety-day mortality.
2. Transplant-free survival.
3. The development of a second episode of OHE within the first 3 months after covered TIPS placement.
4. The development of OHE between 3 and 12 months after covered TIPS placement.
5. Time to development OHE or MHE episode(s).
6. The change in PHES and S-ANT1 test during the study: at time points week 4, week 12 and week 52, compared with baseline.
7. The change in Liver Frailty Index score at week 12 and week 52, compared with baseline.
8. Differences in molecular composition of peripheral/portal blood samples at TIPS placement.
9. Differences in molecular composition of peripheral blood samples at baseline, compared with day 10 post-TIPS, week 4, week 12 and week 52.
10. Quality of life.
11. Costs and cost-effectiveness.

## Methods and analysis

### Trial design

**Figure 1:**
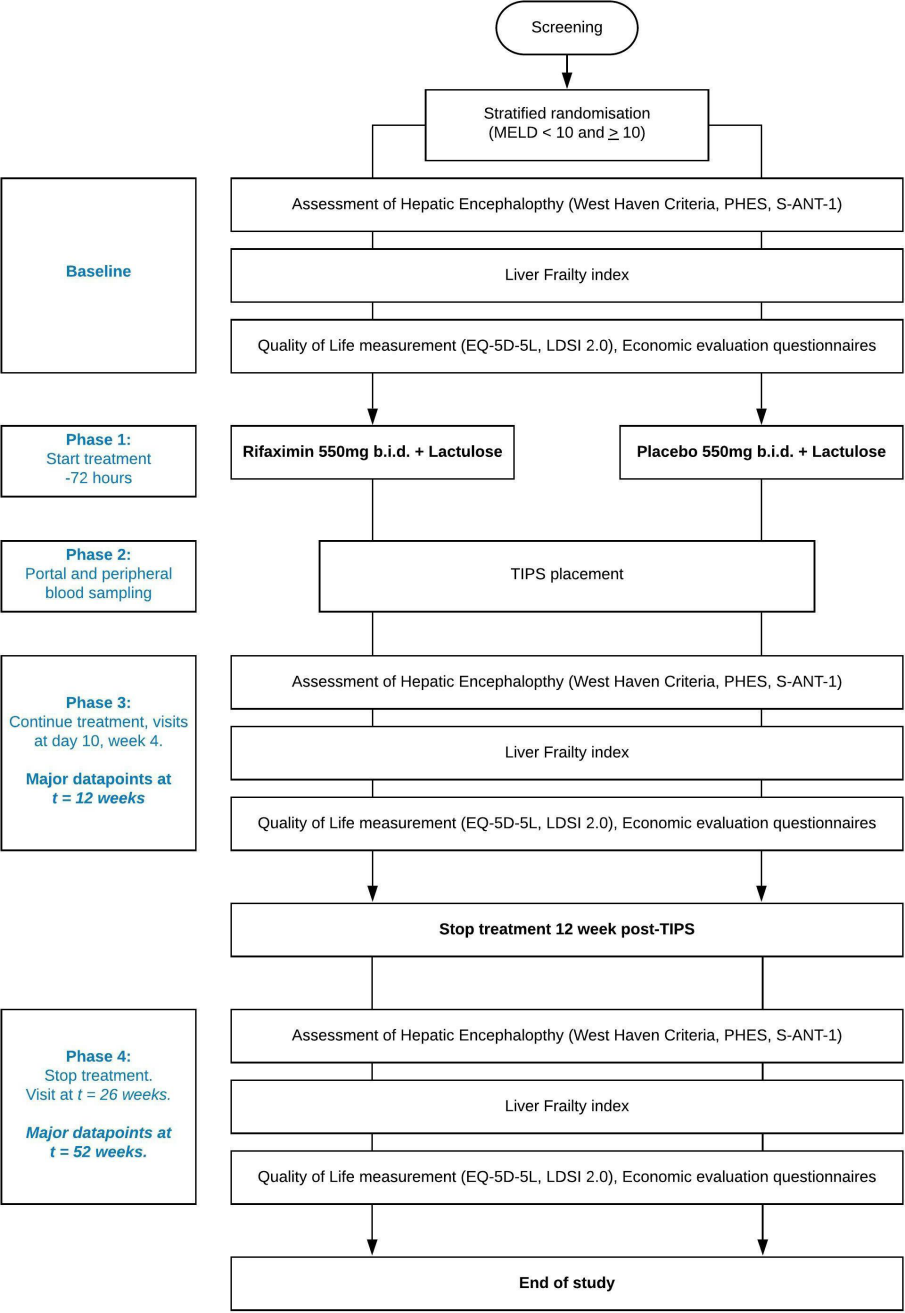
Flowchart of the Prevention of hepatic Encephalopathy by Administration of Rifaximin and Lactulose in patients with liver cirrhosis undergoing placement of a TIPS (PEARL) trial.

The Prevention of hepatic Encephalopathy by Administration of Rifaximin and Lactulose in patients with liver cirrhosis undergoing placement of a TIPS (PEARL) trial, is a multicentre randomised, double blind, placebo controlled trial. Six academic hospitals from the Netherlands and one academic hospital from Belgium will be involved in the recruitment. In order to minimise burden for the study subjects, this trial has been designed in such a way that visits for patients are the same as standard of care. A flowchart of the trial design is found in figure 1.

### Eligibility criteria

#### Inclusion criteria

All of the following criteria must be met in order to be eligible for participation in this trial:

1. Elective-covered TIPS placement for refractory ascites (a) and/or recurrent variceal bleeding (b):
  - Recurrent tense ascites and at least one of the following criteria:
    - Not responding to the maximal dose of diuretics (400 mg spironolactone and 160 mg furosemide).
    - Kidney insufficiency (creatinine >135 μmol/L) induced by diuretics.
    - Electrolyte disturbances (sodium <125 mmol/L, potassium >5.5 mmol/L) induced by diuretics.
    - Not tolerating higher dose of diuretics (eg, because of subjective side effects like muscle cramps).
  - (Recurrent) variceal bleeding, not responsive to treatment with endoscopic band ligation and/or beta-blockers, with a high risk of failure of endoscopic treatment:
    - Patients with a variceal bleeding and Child-Pugh C (10–13 points) cirrhosis or
    - Patients with a variceal bleeding, Child-Pugh B and an active bleeding during endoscopy.
2. Age ≥18 years.
3. Confirmed liver cirrhosis as documented by liver biopsy, elastography (eg, Fibroscan) or combination of usual radiological and biochemical criteria.
4. Signed informed consent.

#### Exclusion criteria

1. Any absolute contraindications for TIPS placement:
  - History of HE grades II–IV without precipitating factor (such as dehydration, variceal bleeding, SBP or other infection).
  - Heart failure New York Heart Association ≥grade 3.
  - Hepatocellular carcinoma (multifocal or large or centrally located).
  - Systemic infection/sepsis.
  - Severe pulmonary hypertension.
  - Unrelieved bile duct obstruction.
  - Technically not feasible.
  - Poor liver function (Model for End-stage Liver Disease (MELD) score >20).
2. Use of ciclosporin (124-fold higher systemic exposure rifaximin).^32^
3. Life-threatening variceal bleeding with emergency TIPS placement that cannot be delayed for 72 hours.
4. Age >80 years.
5. Non-cirrhotic PH.
6. Portal vein thrombosis (main trunk).
7. HIV.
8. Current or recent (<3 months) use of rifaximin.
9. Overt neurologic diseases such as Alzheimer’s disease, Parkinson’s disease.
10. Pregnant or breastfeeding women.

### Randomisation and concealment of allocation

After the screening visit, eligible study subjects will be stratified for MELD score <10 and >10. Study subjects are stratified randomised to the treatment or placebo in a 1:1 ratio. Randomisation with random blocks of sizes 4, 6 or 8 is performed by Castor Electronic Data Capture (Ciwit B.V., The Netherlands) in an automated way and sent to the local clinical trial pharmacy. Neither the treating physician nor the patient is aware of the randomisation result. Deblinding is possible in the case standard of care that might be withheld (see safety paragraph).

### Intervention

Rifaximin 550 mg two times per day will be prescribed, in combination with a starting dose of 25 mL lactulose two times per day and further dependent on the amount of daily bowel movements, with the objective of two soft stools per day. In the case of a study subject already being treated with lactulose, that dose will be used. Intervention will start 72 hours before TIPS placement and will last till 3 months after TIPS placement. The control group will receive placebo in combination with lactulose (as described above).

In the event of lactulose-related diarrhoea, dosage will be lowered to 10 mL two times a day. If diarrhoea persists: dosage is lowered to 5 mL two times a day. Once subjects have no diarrhoea anymore, dosage can be increased, with the objective to achieve two soft stools a day.

### Outcome measures and study procedures

#### Primary outcome

The development of OHE within 3 months after covered TIPS placement is determined by the West Haven criteria.

#### Secondary outcomes

Ninety-day mortality; development of a second episode of OHE within the first 3 months; development of OHE in the period between 3 and 12 months after covered TIPS placement; development of MHE between covered TIPS placement and 12 months after placement; time to development of OHE or MHE episodes; increase in the PHES, S-ANT1 score and LFI compared with baseline. Difference in the composition of portal blood samples, drawn at TIPS placement. TIPS placement data will be recorded. Furthermore, quality of life will be assessed by the Liver Disease Symptom Index V.2.0 (LDSI V.2.0) and EQ-5D-5L questionnaires. Costs will include costs of healthcare, productivity loss due to sick leave from work and out-of-pocket expenses.

### Assessment schedule

A detailed assessment schedule is found in table 1.

**Table 1:** Time and events schedule—PEARL trial

Procedure	Screening	Day 7 (baseline)	Day 3	Day 0 TIPS	Day 10	W4	W12	W26	W52
**Window (days**)	max - 21	±4			±7	±7	±7	±7	±7
**Eligibility assessments**									
Informed consent	X								
Inclusion/exclusion criteria	X								
Medical history +concomitant medication	X								
**Study medication**			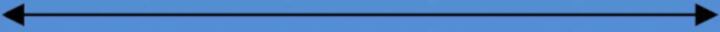						
**Assessments**									
Physical examination*	X	X			X	X	X	X	X
Liver frailty index		X					X		X
Assessment of hepatic encephalopathy by West Haven criteria	X	X			X	X	X	X	X
Assessment of hepatic encephalopathy by PHES and S-ANT1		X				X	X		X
**Assessment of quality of life†**		X					X		X
**Economic evaluation questionnaires‡**		X					X		X
Assessment of adverse events				X	X	X	X	X	X
Assessment of compliance and concomitant medication				X	X	X	X	X	X
**Laboratory tests**§	X	X			X	X	X		X
Storage of peripheral blood and/or portal (*) vein blood		X		X*			X		X
Storage of stool sample		X		X			X		X
Storage of saliva samples		X		X			X		X
Radiological assessment and flow measurement	X				X		X		X

NB: Visit day 7 might be the same as the day of start study medication.

*Height and weight, vital signs, including pulse rate, blood pressure, respiration rate and temperature, signs of ascites or oedema.

†EQ-5D-5L questionnaire and LDSI 2.0 questionnaire.

‡Adapted version of the Productivity Cost Questionnaire and Medical Consumption Questionnaire.

§Clinical chemistry: sodium, potassium, serum creatinine, glucose, alanine aminotransferase, aspartate aminotransferase, total and direct bilirubin, alkaline phosphatase, gamma-GT, C reactive protein, albumin, ammonia. Coagulation: international normalised ratio, PT. haematology: haematocrit, haemoglobin.

PHES, Psychometric Hepatic Encephalopathy Score; S-ANT1, simplified one min animal naming test.

### HE assessment

HE will be assessed by West Haven criteria during all study visits. Additionally, HE is assessed by PHES and S-ANT1 at baseline, week 4, week 12 and week 52.

### Frailty assessment

Liver Frailty Index test will be performed at baseline, week 12 and week 52.

### TIPS placement data

The following data of TIPS placement will be recorded: intravenous antibiotic prophylaxis, method of anaesthesia, per procedural medication, mean ventilation pressure, TIPS placement technique, length of puncture canal, type of covered stent (Viatorr or Viatorr Controlled Expansion), length, diameter and dilatation of this stent, duration of the procedure, visualised collaterals, pressure measurements, embolisation and registration of possible complication(s).

### Quality of life and economic evaluation questionnaires

Quality of life questionnaires are evaluated by EQ-5D-5L and LDSI V.2.0 at baseline, week 12 and week 52. Economic evaluation questionnaires to assess healthcare used and productivity losses are handed out as well at these time points.

### Peripheral and portal blood samples

As shown in table 1, peripheral and portal blood samples will be taken from study subjects at several time points. Patients will be requested to fast 12 hours before blood withdrawal in order to avoid interference with, for example, dietary components in the analysis. Samples of peripheral blood at baseline will be compared with the samples at week 12 and week 52. Samples of peripheral blood (4 hours - 5 min before TIPS placement) will be compared with portal blood samples. Portal samples will be withdrawn directly after TIPS placement using a catheter. When there are clinical signs that a reintervention of the TIPS is inevitable, additional portal and peripheral blood samples will be withdrawn. Additional sampling of portal blood can only occur with a maximum of three times during the first 3 months. In the case that reintervention is necessary after 3 months, another two additional times of sampling are possible in the study period between 3 and 12 months after TIPS placement.

Untargeted metabolomics will be used to study differences in peripheral and portal blood samples as well to study differences in peripheral blood samples from the different time points. Moreover, lipidomics and small molecule analyses will be performed, all using liquid chromatography with tandem mass spectrometry. A more targeted approach is dependent on the results of these experiments.

### Saliva and stool samples

For two ancillary studies, saliva and stool samples are collected for microbiome analyses in some participating centres (due to logistical reasons and a smaller sample size) at baseline, day 0, week 12 and week 52.

### Statistical considerations

#### Sample size

The incidence of post-TIPS OHE is 20%–54% in international studies.^7–10^ To determine the incidence of new onset or worsening HE in our own population, we performed a retrospective analysis of the last 16 years of patients undergoing TIPS placement.^33^ This study revealed that approximately 33% of the patients suffered from new or worsening of existing HE within 30 days after TIPS placement. Between 30 and 90 days after TIPS placement, 17% still had HE. Actual percentages might be higher due to the retrospective nature of the study. Reduction of HE is achieved in 58% of the patients.^27^ However, due to selection bias, we estimate that in our study population. 50% reduction is realistic. The calculation of the sample size (nQuery Advisor V.7.0) was done for a χ^2^ test. Assuming that the effect of rifaximin and lactulose is a 50% reduction of HE, incidence of HE is expected to drop from 33% to 16.5%. With a two-sided 5% alpha, power of 80%, a total of 107 study subjects are needed in both groups. With an estimated dropout of 10%, a total of 119 study subjects are needed in the intervention group and 119 study subjects in the control group.

### Analysis of outcome measures

A comprehensive statistical analysis plan (SAP) will be provided separately. Data will be analysed according to the intention to treat principle. Descriptive methods will be used to assess quality of data, homogeneity of treatment groups and endpoints. A p value <0.05 is considered statistically significant. A brief outline is given in the next paragraph.

#### Primary outcome measurement

The primary outcome, development of OHE within 3 months after TIPS placement determined by the West Haven criteria, will be compared between the intervention and placebo group. Percentage differences with corresponding 95% CI will be reported as absolute improvement in percentages.

#### Secondary outcomes

Secondary outcomes will be analysed using either a t-test or Mann-Whitney U test for continuous data or a χ^2^ test for categorical data, as appropriate. Kaplan-Meier curves will be used to determine transplant-free survival. Kaplan-Meier curves will be used to determine the length of time for the subjects to reach the primary or secondary endpoint with regards to OHE. Censoring will be applied in analysis for liver transplantation or death by any cause. When censoring is applied, Cox proportional hazards model will be used. Comparison of peripheral and portal blood samples at TIPS placement will be analysed using a Wilcoxon signed rank test. Repeatedly measured endpoints will be analysed with mixed models, for the time point(s) they are measured (see table 1).

### Economic evaluation

On the condition that the outcomes of this trial show beneficial results, a cost-effectiveness analysis (CEA), cost-utility analysis (CUA) and budget impact analysis (BIA) will be performed. Costs per patient without post-TIPS HE and the costs per quality-adjusted life year (QALY) will be used as primary economic outcomes. The CEA will estimate costs per additional patient without post-TIPS HE, offset against cost savings associated with reduced incidence of post-TIPS HE by the use of rifaximin and lactulose for a short-term horizon (12 months). The CUA will estimate costs per additional QALY, with a horizon of 5 years. Analyses for the short-term horizon will be based on the empirical data from this trial. Observed differences in QALYs as well as resource use/costs will be extrapolated to 5 years evaluating medium-term consequences. A longer horizon is not reasonable, since patients with end-stage liver cirrhosis have a poor long-term survival or will be transplanted after further progression of disease. Resource use during admission(s) will be assessed by case report form, complemented with the institute for Medical Technology Assessment Medical Consumption Questionnaire, Productivity Costs Questionnaire and patients’ health records during follow-up. To estimate unit costs for observed volumes of resource use, Dutch reference prices will be used. Health state utilities to estimate QALYs will be derived from EQ-5D-5L measurements at baseline, after 12 weeks of treatment and 9 months after cessation of rifaximin and lactulose. Finally, a BIA from a governmental and health insurer perspective will be performed, describing the financial consequences of prophylactic use of rifaximin and lactulose and reduced number of hospital admissions for the extramural drugs budget respectively budget for specialised healthcare. The BIA will be performed in accordance to the Dutch guideline for economic evaluations in healthcare, using the international accepted International Society for Pharmacoeconomics and Outcomes Research (ISPOR) principles.

### Missing data

All efforts will be made to collect all data for each study subject to minimise missing data points. In the case of missing data, a multiple imputation approach will be applied, to be further detailed in the SAP.

### Training and monitoring

Study staff members at participating sites are trained during an initiation visit to ensure that study procedures are carried out uniformly. The study is monitored by the Clinical Research Unit of the Amsterdam University Medical Centres. A full monitoring plan is available. Multiple site visits will take place for source data verification and to verify adherence to the study protocol and its procedures.

### Safety

Both investigational medicinal products used in this trial are safe and effective for (cirrhotic) patients and widely used. Known side effects of lactulose are abdominal bloating and diarrhoea. These side effects are highly dosage dependent. Lactulose should therefore be initially administered two times per day 25 mL, or in case of a study subject already is being treated with lactulose, that dose will be used to minimise side effects. If lactulose-related side effects occur, a step-down approach is in place to minimise these unwanted side effects, as described in the intervention paragraph.

In the case that OHE grade 3 (somnolent but responsive to verbal stimuli) occurs for the first time, patients will be treated with standard care (eg, increase of lactulose), as add-on to the study medication.

In the case that OHE grade 4 (coma) occurs, study medication will be temporarily discontinued and restarted as soon as possible after recovery of the HE episode.

In the case that a patient develops a second episode of OHE, allocation will be deblinded since patients will otherwise might be withheld from standard of care.

## Ethics and dissemination

### Ethical considerations

The study protocol was peer-reviewed by external reviewers during the grant application. In the Netherlands, the study protocol was reviewed and approved by the Medical Research Ethics Committee of the Academic Medical Centre, Amsterdam (2018-332#B2019406) and competent authority The Central Committee on Research involving Human Subjects (NL68205.018.18). In Belgium, this study was reviewed and approved by the Ethics Committee Research UZ/KU Leuven (S62577) and competent authority The Federal Agency for Medicines and Health Products (U89922A). This study will be conducted in accordance with Good Clinical Practice guidelines and the principles of the Declaration of Helsinki. This study is registered in ClinicalTrials.gov (NCT04073290) and EudraCT database (2018-004323-37).

### Dissemination

Results of this RCT will be submitted for presentation at (inter)national congresses and publication in a peer-reviewed journal. The coordinating investigator will prepare the manuscript and authorship is determined by the publication policy as stated in the study protocol. A lay summary of the study results will be made available through the website of the funder, the Netherlands Organisation for Health Research and Development (ZonMw). This summary will also be published in the magazine and website of the Dutch Liver Patients Association.
